# Burden of Illness and Quality of Life in Tuberous Sclerosis Complex: Findings From the TOSCA Study

**DOI:** 10.3389/fneur.2020.00904

**Published:** 2020-08-28

**Authors:** Anna C. Jansen, Stephanie Vanclooster, Petrus J. de Vries, Carla Fladrowski, Guillaume Beaure d'Augères, Tom Carter, Elena Belousova, Mirjana P. Benedik, Vincent Cottin, Paolo Curatolo, Maria Dahlin, Lisa D'Amato, José C. Ferreira, Martha Feucht, Christoph Hertzberg, Sergiusz Jozwiak, John A. Lawson, Alfons Macaya, Ruben Marques, Rima Nabbout, Finbar O'Callaghan, Jiong Qin, Valentin Sander, Matthias Sauter, Seema Shah, Yukitoshi Takahashi, Renaud Touraine, Sotiris Youroukos, Bernard Zonnenberg, J. Chris Kingswood

**Affiliations:** ^1^Pediatric Neurology Unit, Department of Pediatrics, UZ Brussel VUB, Brussels, Belgium; ^2^Department of Public Health, Vrije Universiteit Brussel, Brussels, Belgium; ^3^Division of Child and Adolescent Psychiatry, University of Cape Town, Cape Town, South Africa; ^4^Associazione Sclerosi Tuberosa ONLUS, Milan, Italy; ^5^European Tuberous Sclerosis Complex Association, In den Birken, Dattein, Germany; ^6^Association Sclérose Tubéreuse de Bourneville, Gradignan, France; ^7^TSA Tuberous Sclerosis Association, Nottingham, United Kingdom; ^8^Research and Clinical Institute of Pediatrics, Pirogov Russian National Research Medical University, Moscow, Russia; ^9^SPS Pediatrična Klinika, Ljubljana, Slovenia; ^10^Hôpital Louis Pradel, Claude Bernard University Lyon 1, Lyon, France; ^11^Tor Vergata University Hospital, Rome, Italy; ^12^Karolinska University Hospital, Stockholm, Sweden; ^13^Novartis Farma S.p.A., Origgio, Italy; ^14^Centro Hospitalar Lisboa Ocidental, Lisbon, Portugal; ^15^Department of Pediatrics and Adolescent Medicine, Medical University Vienna, Affiliated Partner of the ERN EpiCARE, Vienna, Austria; ^16^Vivantes-Klinikum Neukölln, Berlin, Germany; ^17^Department of Child Neurology, Medical University of Warsaw, Warsaw, Poland; ^18^Department of Neurology and Epileptology, The Children's Memorial Health Institute, Warsaw, Poland; ^19^The Tuberous Sclerosis Multidisciplinary Management Clinic, Sydney Children's Hospital, Randwick, NSW, Australia; ^20^Hospital Universitari Vall d'Hebron, Barcelona, Spain; ^21^Institute of Biomedicine (IBIOMED), University of Leon, León, Spain; ^22^Department of Pediatric Neurology, Necker Enfants Malades Hospital, Paris Descartes University, Paris, France; ^23^Institute of Child Health, University College London, London, United Kingdom; ^24^Department of Pediatrics, Peking University People's Hospital (PKUPH), Beijing, China; ^25^Tallinn Children Hospital, Tallinn, Estonia; ^26^Klinikverbund Kempten-Oberallgäu gGmbH, Kempten, Germany; ^27^Novartis Healthcare Pvt Ltd, Hyderabad, India; ^28^National Epilepsy Center, Shizuoka Institute of Epilepsy and Neurological Disorders, Shizuoka, Japan; ^29^Department of Genetics, CHU-Hôpital Nord, Saint Etienne, France; ^30^St. Sophia Children's Hospital, Athens, Greece; ^31^University Medical Center, Utrecht, Netherlands; ^32^Cardiology Clinical Academic Group, Molecular and Clinical Sciences Research Centre, St Georges University of London, London, United Kingdom

**Keywords:** tuberous sclerosis complex, quality of life, burden of illness, epilepsy, TOSCA

## Abstract

Research on tuberous sclerosis complex (TSC) to date has focused mainly on the physical manifestations of the disease. In contrast, the psychosocial impact of TSC has received far less attention. The aim of this study was therefore to examine the impact of TSC on health, quality of life (QoL), and psychosocial well-being of individuals with TSC and their families. Questionnaires with disease-specific questions on burden of illness (BOI) and validated QoL questionnaires were used. After completion of additional informed consent, we included 143 individuals who participated in the TOSCA (TuberOus SClerosis registry to increase disease Awareness) study. Our results highlighted the substantial burden of TSC on the personal lives of individuals with TSC and their families. Nearly half of the patients experienced negative progress in their education or career due to TSC (42.1%), as well as many of their caregivers (17.6% employed; 58.8% unemployed). Most caregivers (76.5%) indicated that TSC affected family life, and social and working relationships. Further, well-coordinated care was lacking: a smooth transition from pediatric to adult care was mentioned by only 36.8% of adult patients, and financial, social, and psychological support in 21.1, 0, and 7.9%, respectively. In addition, the moderate rates of pain/discomfort (35%) and anxiety/depression (43.4%) reported across all ages and levels of disease demonstrate the high BOI and low QoL in this vulnerable population.

## Introduction

Tuberous sclerosis complex (TSC) is a multi-system genetic disorder with a global incidence of 1 per 6,000–10,000 live births. Over a million people are estimated to be affected worldwide (1). It is characterised by growth of benign tumours in various organs throughout the body, including the brain, kidney, lungs, and skin (2). It is also associated with behavioural, psychiatric, intellectual, academic, neuropsychological, and psychosocial difficulties, grouped under the umbrella term TAND (TSC-Associated Neuropsychiatric Disorders) (3, 4). The clinical presentation of TSC manifestations is complex (5–8). Its natural course varies between individuals, with symptoms occurring at variable ages and severity ranging from very mild to severe, which may even lead to death. Furthermore, individuals with TSC are expected to have lifelong follow-up care to ensure the early detection of potentially life-threatening complications. The diverse clinical presentation represents significant disease, healthcare, and treatment burden (9).

To date, the majority of TSC research has concentrated on the pathophysiology, epidemiology, diagnosis, and treatment of the condition (10). Relatively little has been done to evaluate the impact of TSC on the quality of life (QoL) and social well-being of individuals with TSC and their families. A number of researchers have focused on the burden of specific aspects of TSC, such as epilepsy, subependymal giant cell astrocytoma (SEGA), facial angiofibroma, and renal angiomyolipoma (8, 9, 11–14). Others have evaluated the impact of specific treatments on QoL such as following epilepsy surgery (15), or have studied specific groups such as the impact on adult caregivers (10, 16). A retrospective study that evaluated parents of 99 children with TSC showed that about 50% reported clinically significant parental stress. The stress was related to the presence of current seizures, a history of psychiatric diagnosis, intellectual disability, and/or behavioural problems in the children (17). A web-based United Kingdom (UK) survey of individuals with TSC and their caregivers showed significantly lower health state utility values (HSUVs) compared with the general population reference value for the UK value set of the three-level version of the EuroQol-5D (EQ-5D-3L). This indicates substantial impairment in individuals with TSC (18). Zöllner et al. performed a systematic review on the burden of illness (BOI) in TSC and included 33 articles published up to October 2019, only 14 of which addressed QoL (19). We sought to assess the impact of TSC on the lives of individuals or their caregivers in terms of BOI and QoL, using a combination of ancillary disease-specific questions on BOI and validated QoL questionnaires in seven European countries.

## Methods

TOSCA, a natural history registry in TSC, was conducted in 170 sites across 31 countries worldwide. A detailed description of the methods of the TOSCA study has been provided previously (20). The registry consists of a “core” section and six “petals” or “research projects”. Here, we present findings from one of the research projects focusing on BOI and QoL in individuals with TSC.

### Participants

Selection of countries participating in this research project was based on the availability of the validated QoL questionnaires in the primary language used in that country. Based on this criterion, TSC individuals of any age from seven European countries were eligible for this specific research project, after signing an additional consent form.

### Measuring Burden of Illness

All enrolled individuals were asked to complete a set of ancillary questions addressing social care needs (circumstances of living arrangements, financial, social, and psychological support, and information sources), healthcare needs (health insurance, medical care and level of satisfaction, genetic testing, and genetic counselling), impact on education and employment, impact on family, and transition from paediatric to adult care (Supplementary Material). These ancillary questions were developed by patient representatives, who were part of the TOSCA Working Committee in collaboration with the TSC patient associations. Draft questionnaires were reviewed by two caregivers for clarity and comprehensiveness. When individuals were unable to complete the questionnaires by themselves, caregivers were asked to complete the proxy version of the questionnaires (caregiver report).

### Measuring Quality of Life

For evaluating QoL, validated questionnaires in local languages were administered to individuals with TSC/caregivers who participated in this research project. These included the following: (1) EuroQol-5D (EQ-5D), a self-complete questionnaire for adults (age, ≥18 years); the EQ-5D proxy version 1 was completed by the caregiver for children or adolescents for adults who were unable to complete the report by themselves; (2) QoL in Epilepsy Inventory-31-Problems (QOLIE-31)-P for adults (age, ≥18 years) with epilepsy, completed by the individuals themselves; (3) QoL in Childhood Epilepsy (QOLCE) for children <10 years old with epilepsy (completed by caregivers); (4) QoL in Epilepsy Inventory for Adolescents-48 (QOLIE-AD-48) for children aged 11–17 years with epilepsy, completed by the subjects themselves.

### Data Analyses

Data on QoL and BOI were recorded once (i.e., no follow up requested) before the data cut-off date (10 August, 2017). A copy of the collected paper questionnaires was sent from each clinical site to the clinical research organization (CRO) for data entry in the TOSCA study. Data were then extracted and analysed by the CRO. Responses to the BOI questions and QOL scales were summarised by descriptive statistics (number of responders, mean, standard deviation, median, range, frequency), considering age-based subgroup as children (<11 years), adolescents (age 11 to <18 years) and adults (age ≥18 years).

Individuals with TSC or their caregivers, rated their level of impairment across five dimensions (mobility, self-care, usual activities, pain/discomfort, and anxiety/depression). Each dimension has three levels: no problems, some problem and confined to bed. The mean thermometer score for EQ-5D and mean health state score for QOLIE-31-P questionnaire were recorded on a scale from 0 to 100, with 0 being the worst health state imaginable and 100 the best. Furthermore, each patient rated the importance of the seven QOLIE 31-P sub scales (energy, mood, daily activities, cognition, medication effects, seizure worry, and overall quality of life) from one to seven, with one being the most important topic and seven the least important one. The sub-scale scores of QOLIE-31-P questionnaire were the means of the converted item scores multiplied by the distress score. The total QOLIE-31-P score was calculated by dividing the sum of the sub scales by the sum of the distress scores multiplied by 100. If more than half the items in a sub-scale had not answered, the sub-scale was not included in the total score. For each sub scale of QOLCE, the answer for each item was converted to a 0 to 100 point score, where high scores reflect the highest level of functioning.

## Results

Hundred fouty three individuals (88 children and adolescents, and 55 adults) from seven European countries were enrolled in this research project as part of the TOSCA study (Table 1). The mean time since initial diagnosis of TSC was 13.5 years (median, 11.2 years; range, 1.6–43.5). Of the 143 individuals enrolled, 67 (28 adults) had epilepsy (46.9%). The mean duration of epilepsy was 16.6 years (median, 12.8 years; range, 2.7–55.4).

**Table 1:** Demographic characteristics.

	**Overall (*N* = 143)**
**Sex #**	
Male	54 (37.8)
Female	88 (61.5)
**Age at consent (years)**	
n	142
Mean (SD)	19.8 (15.24)
Median (range)	14 (3–72)
**Duration of TSC (years)**	
n	141
Mean (SD)	13.5 (9.44)
Median (range)	11.2 (1.6–43.5)
**Country**	
Belgium	24 (16.8)
France	30 (21.0)
Germany	11 (7.7)
Italy	58 (40.6)
Spain	11 (7.7)
Sweden	6 (4.2)
UK	3 (2.1)
**Individuals with epilepsy**	
n(%)	67 (46.9)
Duration of epilepsy (years) at start of research project	
*n*	66
Mean (SD)	16.6 (12.53)
Median (range)	12.8 (2.7–55.4)

# *Information on sex was not available for 1 patient. Values are expressed as n (%) unless otherwise stated*.

### Burden of Illness: Self-Reported Outcomes

17 adolescents (19.3%; aged between 11 and <18 years) and 38 adults (69.1%) completed the questionnaire independently. Of these, one (5.9%) adolescent and five adults (13.2%) needed extra assistance at home. In most cases, assistance was provided by unpaid caregivers (a family member or friend). 29.4% adolescents and 42.1% of adults felt that assistance and support at home was not sufficient (Table 2). Financial, social, and psychological support was received by 8 (21.1%), 0 (0%), and 3 (7.9%) of adult respondent, respectively.

**Table 2:** Social care needs: self- and caregiver-reported outcomes.

	**Self-reported individuals with TSC**		**Individuals with TSC reported by caregivers**	
	**Adolescents *N* = 17**	**Adults *N* = 38**	**Children/Adolescents *N* = 71**	**Adults *N* = 17**
**Circumstances of living arrangements**				
Lives alone	NA	5 (13.2)	NA	1 (5.9)
Lives with spouse/partner	NA	21 (55.3)	NA	2 (11.8)
Lives with other family	NA	10 (26.3)	NA	13 (76.5)
Information missing	NA	2 (5.3)	NA	1 (5.9)
**Help with daily activities needed**				
Yes	NA	3 (7.9)	NA	8 (47.1)
No	NA	35 (92.1)	NA	9 (52.9)
**Assistance at home**				
Nurse	0	0	1 (1.4)	1 (5.9)
Daily assistance by professional carer (paid)	1 (5.9)	0	5 (7.0)	1 (5.9)
Caregiver assistance from friend/family/relative (not paid)	0	5 (13.2)	16 (22.5)	7 (41.2)
Individuals felt that assistance and support at home was not sufficient	5 (29.4)	16 (42.1)	31 (43.7)	6 (35.3)
**Financial, social, and psychological support**				
Disability allowance	6 (35.3)	8 (21.1)	39 (54.9)	13 (76.5)
Caregiver allowance	1 (5.9)	0	9 (12.7)	0
Social worker assistance	1 (5.9)	0	6 (8.5)	1 (5.9)
Social services support	1 (5.9)	0	3 (4.2)	2 (11.8)
Psychological counselling	2 (11.8)	3 (7.9)	10 (14.1)	0
**Used sources for information about rights and benefits**				
Physician	7 (41.2)	25 (65.8)	46 (64.8)	9 (52.9)
Internet/Websites	9 (52.9)	14 (36.8)	49 (69.0)	7 (41.2)
Patient group	2 (11.8)	5 (13.2)	20 (28.2)	6 (35.3)
Social worker	1 (5.9)	1 (2.6)	21 (29.6)	3 (17.6)
Local government	1 (5.9)	4 (10.5)	6 (8.5)	2 (11.8)
Nurse	0	2 (5.3)	3 (4.2)	3 (17.6)
**Most useful source**				
Physician	9 (52.9)	25 (65.8)	34 (47.9)	8 (47.1)
Internet/Websites	4 (23.5)	4 (10.5)	19 (26.8)	3 (17.6)
Patient group	2 (11.8)	2 (5.3)	12 (16.9)	4 (23.5)
Social worker	1 (5.9)	0	13 (18.3)	3 (17.6)
Local government	1 (5.9)	2 (5.3)	3 (4.2)	0
Nurse	0	0	1 (1.4)	0

*NA not applicable. Values are expressed as n (%)*.

**Figure 1:**
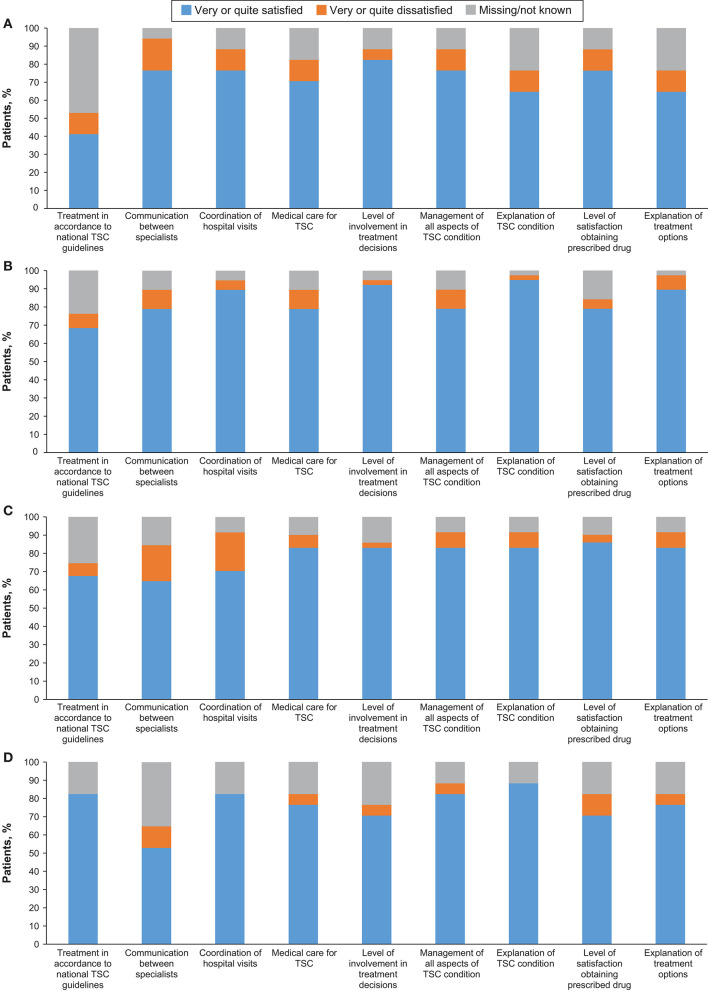
Satisfaction with treatment aspects in **(A)** Self-reported children, **(B)** Self-reported adults, **(C)** Caregiver-reported children, and **(D)** Caregiver-reported adults.

Nine adolescents (52.9%) and 16 adults (42.1%) had access to public and/or private insurance (Table 3). Although none of the individuals reported that they had to pay extra for private insurance due to TSC, two adults (5.3%) reported that health or any kind of insurance was denied due to TSC. TSC was managed by TSC specialists in 12 adolescents (70.6%) and 28 adults (73.7%). Twenty-nine adults (76.3%) reported that they had access to a TSC clinic when required, while no access to TSC clinics were reported by six adults (15.8%). TSC was managed by more than three physicians in 15 adults (39.5%). Smooth transition from paediatric to adult care was reported by only 14 adults (36.8%). Nearly one fifth of patients were dissatisfied with various aspects of their medical care and nearly 50% were not able to report if their care followed clinical guidelines (Figure 1).

**Table 3:** Health care needs: self- and caregiver-reported outcomes.

	**Self-reported individuals with TSC**		**Caregivers-reported individuals with TSC**	
	**Adolescents *N* = 17**	**Adults *N* = 38**	**Children/adolescents *N* = 71**	**Adults *N* = 17**
**Individuals with health insurance**				
Private insurance	2 (11.8)	7 (18.4)	31 (43.7)	6 (35.3)
Public insurance	6 (35.3)	14 (36.8)	37 (52.1)	4 (23.5)
No insurance	7 (41.2)	15 (39.5)	11 (15.5)	8 (47.1)
Individuals thought to have paid extra for private insurance due to TSC condition	0	0	1 (1.4)	0
Public insurance was denied due to TSC	0	2 (5.3)	9 (12.7)	1 (5.9)
**Genetic testing**				
Patient had genetic testing for TSC	13 (76.5)	31 (81.6)	57 (80.3)	16 (94.1)
Patient was offered genetic testing but did not do it	1 (5.9)	1 (2.6)	3 (4.2)	0
Patient had not been offered genetic testing for TSC	0	3 (7.9)	7 (9.9)	1 (5.9)
**Genetic counselling**				
Patient had genetic counselling	9 (52.9)	26 (68.4)	43 (60.6)	10 (58.8)
Patient was offered genetic counselling but decided not to have it	0	0	3 (4.2)	0
Patient had not been offered genetic counselling for TSC	4 (23.5)	6 (15.8)	19 (26.8)	4 (23.5)
**Number of doctors managing TSC**				
1	8 (47.1)	12 (31.6)	17 (23.9)	6 (35.3)
2	3 (17.6)	5 (13.2)	11 (15.5)	1 (5.9)
3	0	3 (7.9)	11 (15.5)	2 (11.8)
>3	6 (35.3)	15 (39.5)	31 (43.7)	7 (41.2)
Data not provided	0	3 (7.9)	1 (1.4)	1 (5.9)
TSC is managed by^*^				
General practitioner/family doctor	1 (5.9)	9 (23.7)	17 (23.9)	6 (35.3)
TSC specialist	12 (70.6)	28 (73.7)	39 (54.9)	16 (94.1)
Other specialist	7 (41.2)	19 (50.0)	49 (69.0)	7 (41.2)
**Access to TSC clinic**				
Individuals had access to clinic when required	13 (76.5)	29 (76.3)	43 (60.6)	16 (94.1)
Distance to TSC clinic from home				
<50 km	10 (58.8)	14 (36.8)	18 (25.4)	4 (23.5)
>50 km	3 (17.6)	15 (39.5)	30 (42.3)	12 (70.6)
**Individuals in contact with national TSC association**				
Yes	9 (52.9)	14 (36.8)	36 (50.7)	9 (52.9)
No	7 (41.2)	22 (57.9)	33 (46.5)	7 (41.2)
Data not available	1 (5.9)	2 (5.3)	2 (2.8)	1 (5.9)

* *Participants may have provided more than one answer. Values are expressed as n (%)*.

TSC was reported to have impacted the career/education progress in three adolescents (17.6%; Table 4). Fourteen adolescents (82.4%) were in mainstream education. Six adolescents (35.3%) received additional support in class; no adolescents were home-schooled. Of the 38 adults, 20 (52.6%) were employed and seven were not able to work (4 due to TSC; 3 due to other reasons). Sixteen adults (42.1%) expressed that TSC had affected their career or education in different ways: impact on career progression/promotions (25%), choice of career (25%), loss of employment (31.3%), part-time rather than full-time work (31.3%), or attainment of education level (37.5%).

**Table 4:** Impact of TSC on education, employment and relationships.

	**Self-reported individuals with TSC**		**Caregivers-reported individuals with TSC**	
	**Adolescents *N* = 17**	**Adults *N* = 38**	**Children/adolescents *N* = 71**	**Adults *N* = 17**
**Impact on education**				
Impact of TSC on career/education of self or caregivers (in case of children)^a^	3 (17.6)	16 (42.1)	47 (66.2)	12 (70.6)
Career progression/promotions	0	4 (25.0)	17 (36.2)	1 (8.3)
Choice of career	0	4 (25.0)	16 (34.0)	1 (8.3)
Loss of employment	2 (66.7)	5 (31.3)	10 (21.2)	1 (8.3)
Part-time work rather than full time	0	5 (31.3)	25 (53.2)	1 (8.3)
Education level attained	1 (33.3)	6 (37.5)	3 (6.4)	10 (83.3)
**Current employment status of self or caregivers (in case of caregiver-reported children)**				
Employed (either full or part-time)	11 (64.7)	20 (52.6)	47 (66.2)	3 (17.6)
Unable to work due to condition	0	4 (10.5)	8 (11.3)	10 (58.8)
Unable to work but not due to condition	0	3 (7.9)	8 (11.3)	1 (5.9)
Student	2 (11.8)	2 (5.3)	0	1 (5.9)
Homemaker	4 (23.5)	7 (18.4)	10 (14.1)	1 (5.9)
**Impact of TSC on relationships of self or caregivers (in case of caregiver-reported children)**				
Family relationships	3 (17.6)	8 (21.1)	29 (40.8)	4 (23.5)
Social relationships	2 (11.8)	14 (36.8)	36 (50.7)	11 (64.7)
Working colleague relationships	0	4 (10.5)	17 (23.9)	1 (5.9)
Child is in mainstream education	14 (82.4)	NA	43 (60.6)	NA
Child receives additional support in class	6 (35.3)	NA	31 (43.7)	NA
Additional support causes child additional problems	2 (11.8)	NA	13 (18.3)	NA

a *Individuals may have reported one or more ways of impact of career/education. Values are expressed as n (%)*.

### Burden of Illness: Caregiver-Reported Outcomes

Parents/Caregivers completed the questionnaires for 71 children and adolescents (80.7%; 38 girls and 32 boys) and 17 adults (30.9%; 11 female and 6 male) who were unable to complete the questionnaires by themselves.

Of the 71 caregiver-reported children and adolescents, 20 (28.2%) needed help at home, provided mainly by unpaid caregivers in 80% of cases (Table 2). Of the 17 caregiver-reported adults, one (5.9%) was living alone, two (11.8%) with a partner, and 13 (76.5%) with other family members. Eight (47.1%) individuals needed help with daily activities. About half of the caregiver-reported individuals (50.7% children and adolescents, and 52.9% adults) were in contact with their local TSC associations.

TSC was managed by TSC specialists in 39 (54.9%) caregiver-reported children and adolescents, and 16 (94.1%) caregiver-reported adults (Table 3). Twenty-three caregivers (32.4%) reported that their children and adolescents did not have access to TSC specialist clinics but most caregiver-reported adults (94.1%) did. Most caregiver-reported children and adolescents (80.3%) and caregiver-reported adults (94.1%) received genetic testing for TSC, but genetic counselling was received only by 60.6% of children and adolescents, and 58.8% of adults. None of the six (35.3%) caregiver-reported adults who received private insurance felt that they had to pay extra due to TSC and only one patient (5.9%) reported that health or any kind of insurance was denied due to TSC.

Caregivers have reported that TSC had affected the career or education of their children and adolescents in different ways. These include part-time work rather than full time (53.2%), impact on career progression/promotions (36.2%), choice of career (34.0%), loss of employment (21.2%), impact on educational attainment (6.4%). Of the 17 caregiver-reported adults, only three (17.6%) were employed while 10 (58.8%) were unable to work due to TSC. Ten (83.3%) carer-reported adults reported impact of educational attainment. Relationships of caregivers had been impacted due to child's TSC in 53.5% of cases with impact on the family, social, and working colleague relationships were reported in 29 (40.8%), 36 (50.7%), and 17 (23.9%) cases, respectively. Impact on the family, social and working relationships by TSC condition have been noted in 76.5% of caregiver-reported adults.

### Quality of Life (QoL) in TSC

#### EQ-5D Questionnaire

**Figure 2:**
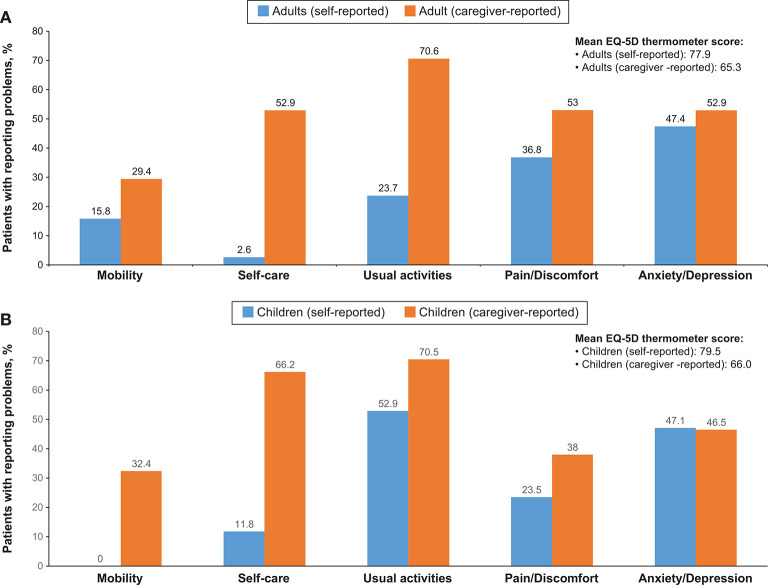
Summary of EQ-5D Questionnaire in **(A)** adults and **(B)** children.

Overall, EQ-5D (or Q-5D proxy version 1) questionnaires were completed for all 143 participants. Difficulty in mobility was reported by 34 individuals (23.8%) and 32 (22.4%) experienced difficulty in self-care. Twenty-six individuals (18.2%) were unable to perform usual activities, fifty individuals (35%) had moderate pain or discomfort and four individuals (2.8%) had extreme pain or discomfort. Sixty-two individuals (43.4%) reported moderate anxiety/depression, while six individuals (4.2%) reported extreme anxiety/depression. Anxiety/depression and pain/discomfort were reported in both self-reported as well as caregiver-reported groups and present in both children and adolescents, and adults (Figure 2). On the thermometer scale of 0–100 (100 being the best state of health imaginable and 0 as worst state imaginable) the mean score was 70.6.

#### QOLIE-31-P Questionnaire

The QOLIE-31-P questionnaire was completed by 24 individuals. The total score of the QOLIE-31-P questionnaire was 71.6 (standard deviation [SD]: ±16.7, Table 5). The mean (±SD) score for different sub-scales were: energy (47.0 ± 27.6), mood (53.4 ± 29.8), daily activities (67.0 ± 33.3), cognition (63.6 ± 37.5), medication effects (56.9 ± 31.5), seizure worry (49.8 ± 31.4), and overall quality of life (53.8 ± 29.1).

**Table 5:** Summary of QOLIE-31-P, QOLCE and QOLIE-AD-48 questionnaire scores.

	***n***	**Mean**	**SD**	**Median**	**Range**
**QOLIE-31-P**					
Energy	24	47.0	27.6	45.0	2.5–90
Mood	24	53.4	29.8	63.5	3.6–92
Daily activities	24	67.0	33.3	73.1	3.8–100
Cognition	24	63.6	37.5	71.1	0.3–100
Medication effects	24	56.9	31.5	57.5	1.3–100
Seizure worry	24	49.8	31.4	45.8	0.4–100
Overall QoL	24	53.8	29.1	58.1	3.3–95
Final Score	24	71.6	16.7	75.8	27.3–93.4
**QOLCE**					
QoL	67	51.5	27.5	50.0	0–100
Physical restrictions	69	44.6	24.4	45.8	0–100
Energy/fatigue	67	54.5	22.7	62.5	0–100
Depression	68	70.7	17.6	75.0	8.3–100
Anxiety	68	58.8	20.5	50.0	25–100
Control/helplessness	64	56.1	20.1	50.0	18.8–100
Self-esteem	65	63.9	19.6	70.0	15–95
Attention/concentration	66	37.5	28.7	32.3	0–100
Memory	58	54.2	23.8	56.3	0–100
Language	60	42.1	28.7	44.4	0–100
Other cognitive functions	64	31.7	29.0	25.0	0–100
Social interactions	56	53.6	21.7	60.0	0–100
Social activities	67	63.8	35.5	66.7	0–100
Stigma	56	66.1	36.4	75.0	0–100
Behaviour	69	50.5	20.6	48.4	0–93.8
General health	68	48.5	27.3	50	0–100
Final score	70	52.3	18.9	51.5	12.2–91.7
**QOLIE-AD-48**					
Epilepsy impact	8	82.7	20.2	91.7	39.6–95.8
Memory/concentration	8	74.1	22.3	82.5	45–100
Physical functioning	8	83.1	18.1	87.5	55–100
Stigma	8	81.9	22.2	83.3	33.3–100
Social support	8	69.5	22.5	59.4	43.8–100
School behaviour	8	97.7	3.2	100.0	93.8–100
Attitudes toward epilepsy	7	30.4	7.6	31.3	18.8–37.5
Health perceptions	8	61.5	9.9	58.3	50.0–75
Final score	7	74.2	13.9	81.2	46.1–85.7

#### QOLCE Questionnaire

The QOLCE questionnaire was completed by 70 caregivers. The mean QOLCE score was 52.3 (SD: ±18.9, Table 5). The mean (±SD) scores of different sub-scales were: QoL (51.5 ± 27.5), physical restrictions (44.6 ± 24.4), energy/fatigue (54.5 ± 22.7), depression (70.7 ± 17.6), anxiety (58.8 ± 20.5), control/helplessness (56.1 ± 20.1), self-esteem (63.9 ± 19.6), attention/concentration (37.5 ± 28.7), memory (54.2 ± 23.8), language (42.1 ± 28.7), other cognitive functions (31.7 ± 29.0), social interactions (53.6 ± 21.7), social activities (63.8 ± 35.5), stigma (66.1 ± 36.4), behaviour (50.5 ± 20.6), and general health (48.5 ± 27.3). The highest score was reported for depression and the lowest for other cognitive functions.

#### QOLIE-AD-48 Questionnaire

Eight adolescents aged 11–17 years with epilepsy completed the questionnaire. The mean total QOLIE-AD-48 questionnaire score was 74.2 (SD: ±13.9, Table 5). The score of the sub-scales were epilepsy impact (82.7 ± 20.2), memory/concentration (74.1 ± 22.3), physical functioning (83.1 ± 18.1), stigma (81.9 ± 22.2), social support (69.5 ± 22.5), school behaviour (97.7 ± 3.2), attitudes toward epilepsy (30.4 ± 7.6), and health perceptions (61.5 ± 9.9).

## Discussion

This study aimed to evaluate BOI and QoL in children and adolescents, and adults with TSC and their families. BOI focused on social care needs, health (care) needs, and impact of TSC on education, employment, and family life. Individuals' QoL was assessed by means of standardized measures of QoL. To our knowledge, this study represented the most comprehensive and multinational evaluation of BOI and QoL in TSC to date.

Four main findings were highlighted by this study. BOI in families with TSC patients was high, as shown by their experiences of insufficient assistance at home and from social services. Individuals with TSC reported significant use of healthcare services but considered the support from TSC associations and patient organizations as inadequate. Also, the impact of TSC on individuals' education, employment, and social and family life was profound. Regarding quality of life, both children and adolescents, and adults reported moderate-to-severe levels of pain or discomfort and anxiety or depression, which was also indicated by their caregivers.

Individuals with TSC and their families have unmet needs with respect to support from social workers who provide various services, corresponding to previous findings (8). Most services were not available, or not offered or performed properly. Possibly, these professionals were insufficiently aware of the specific needs of individuals with or lack the experience to provide appropriate support. Another explanation for this unmet need might be difficulty in reaching out to families of individuals with TSC by social workers due to practical reasons, or families of individuals with TSC had personal barriers to seek help. Clearly, our findings underline the urgent need for increased awareness among social services about the importance of early and systematic follow-up of individuals with TSC and their environment (21). When such needs remain unrecognised, family members feel urged to take on various responsibilities and failed to introduce further professional care in a timely manner, preventing optimal guidance with attention to individual goals or preferences.

Individuals with TSC showed various clinical manifestations for which they visited health specialists. Throughout their lives, they made significant use of healthcare services as a result of the regular multidisciplinary medical care indicated for the management of TSC (22). However, the present study showed that high healthcare utilization and followed-up by a TSC specialist or clinic were unrelated to involvement of TSC associations and patient organizations in the individual's care trajectory. Reasons could be that patients were not familiar with them, not convinced of their significance for their own situation or experience sufficient support from their own private network. It was also plausible that these societal partners failed to reach families with TSC in the right way or did not meet their expectations regarding types of support.

The observed lack of appropriate care services was also reflected in differences between individuals in terms of health insurance, and genetic testing, and counselling. These findings indicate a need for revision and standardization of insurance policies for people with TSC or chronic conditions in general, as well as clinical care characterized by a personalized and transparent approach. Despite this imbalance between care need and care provision, individuals in this study reported satisfaction with how their disease was treated and monitored. Furthermore, the transition from paediatric to adult TSC care was an important area of concern (23). Although this phase is generally considered challenging or difficult (24), our results showed a smooth process in almost half of the cases. Transition-enhancing practices such as use of an individual action plan, implementation of a transition protocol and setting up a mixed paediatric-adult team with a transition coordinator might be useful in TSC care (25, 26).

TSC had a strong influence on the education and professional career of affected individuals. Especially in adults, their level of education, choice of career, career progression and promotion, and employment rate were impacted by the disease. Apart from the presence of TSC, other influences, directly or indirectly, related to the illness should be taken into account. Having few professional expectations for the future, being confronted with negative attitudes of colleagues and lacking arrangements to improve working conditions, might all further reduce the patient's opportunities at work (27, 28). The impact of TSC on education was relatively minor in the group of self-reporting adolescents, a finding that is likely biased by their assumed milder phenotype since they were able to fill-out the BOI and QOL questionnaires independently. Previous research showed a higher degree of absenteeism, impaired performance, and lower productivity at school in paediatric patients (28). It seems therefore advisable to guide young patients on study choice and keep track of adults' working life, while listening to expressed questions, concerns, and problems.

TSC has significant effects on the social well-being and family life of both young and adult individuals with TSC. The patients' high dependence on their environment can lead to feelings of disorientation, loneliness, and clinically significant stress levels in patients, but also in family members (10, 17). Our data show the marked effect of TSC on the income, career, and psychological well-being of the individual's family. Therefore, it is essential to identify and approach the sources of such familial distress, which vary according to the patient's personal characteristics, health status, and living environment. Problems in children and adults with TSC such as severe epilepsy and other persistent health problems, neuropsychiatric disorders (TAND) and a lack of support from the family's network can put a heavy burden on the family of individuals with TSC (2, 29, 30). As a result, the family may become isolated as friendships and professional relationships receive less attention (16). However, it has been shown that external support might help building the family's resources, as they can cope better with the multifaceted problems of TSC and regularly shift their attention from the disease to pleasant events and moments in life (10).

With regard to QoL, moderate to severe levels of pain or discomfort and anxiety or depression were reported by individuals with TSC of all ages as well as by their caregivers. In order to achieve a comprehensive view of health-related QoL in individuals with TSC, research suggested to investigate other indicators such as fatigue, emotional stress, and participation (31). In particular, participation is important, as this multidimensional concept captures how the patient's health determines his or her participation in daily life, taking into account functional and intellectual disabilities. Assessing the individual's participation rate in terms of education, social activities, and leisure time is required for the development of interventions, which enable a long life with a good QoL (32). In future studies on BOI and QoL, standardized instruments to measure participation such as questionnaires for patients and carers could be used (33, 34).

When interpreting the results of this study, certain limitations need to be taken into account. Not all patients completed all questionnaires in the study, and only a small subsample of patients from the TOSCA registry enrolled in the present study. Although the information was collected from both individuals who were able to self-report as well as from caregivers of individuals who were unable to self-report, the overall disease severity of the cohort is likely to be milder compared to that of the global TOSCA registry cohort. Only 46.85% of patients in the current study was reported to have epilepsy in contrast to 83.5% in the overall TOSCA cohort (35). Since epilepsy is known to have a major impact on QoL (36), the burden of illness reported here might reflect the impact at the milder end of the spectrum. Furthermore, these subjects were all recruited from clinicians specialized in TSC care. Therefore, the level of care and satisfaction in the general TSC population is likely to be lower.

Although no data on intellectual ability were collected, 65% of children were following mainstream education. Although school systems differ across countries and attending mainstream education does not imply that children have normal intellectual ability, it seems likely that this reflects again a potential bias towards the milder end of the spectrum. The lack of a personal perspective is another limitation of the study. The questionnaire used to measure BOI contained questions that were developed together with families, which ensures a large patient-oriented input. Although no qualitative research was conducted, a short analysis of the questionnaire's open data fields did confirm the quantified BOI (data not shown).

## Conclusion

Our study confirms the impact of TSC on education, career and social life of patients, and their families. This disease-specific impact is also reflected in patients' quality of life, including moderate-to-high levels of pain or discomfort and anxiety or depression. Unfortunately, despite families' frequent use of healthcare services, provision of well-organized TSC care is not evident as shown by their experiences of insufficient social support and discontinuous pediatric to adult care trajectories. These difficulties further increase the impact on the different life domains of families living with TSC, who would benefit from better coordinated educational, psychosocial, and medical support.

## Data Availability Statement

The datasets presented in this study can be found in online repositories. The names of the repository/repositories and accession number(s) can be found below: Novartis supports the publication of scientifically rigorous analysis that is relevant to patient care, regardless of a positive or negative outcome. Qualified external researchers can request access to anonymized patient-level data, respecting patient informed consent, contacting study sponsor authors. The protocol can be accessed through EnCePP portal http://www.encepp.eu/ (EU PAS Register Number EUPAS3247).

## Ethics Statement

The studies involving human participants were reviewed and approved by independent ethics committee/institutional review board for each centre involved in the study (see Supplementary Materials for full list). Written informed consent to participate in this study was provided by the participants' legal guardian/next of kin.

## Author Contributions

AJ, EB, MB, PC, MD, JF, MF, CH, SJ, JK, JL, AM, RN, VS, MS, RT, and BZ designing the study, patient accrual, clinical care, data interpretation, drafting, revising, final review, and approval of the manuscript. SV data interpretation, drafting, revising, final review, and approval of the manuscript. PV designing the study, data interpretation, drafting, revising, final review, and approval of the manuscript. CF designing the study, data interpretation, drafting, revising, final review, and approval of the manuscript. CF, GB, TC, VC, FO'C, JQ, YT, and SY designing the study, data interpretation, drafting, revising, final review, and approval of the manuscript. LD'A designing the study, trial management, data collection, data analysis, data interpretation, drafting, revising, final review, and approval of the manuscript. RM designing the study, data analysis, data interpretation, drafting, revising, final review, and approval of the manuscript. SS designing the study, trial statistician, data analysis, data interpretation, drafting, revising, final review, and approval of the manuscript. All authors contributed to the article and approved the submitted version.

## Conflict of Interest

AJ, PV, EB, TC, VC, PC, GB, JK, JF, MF, CF, CH, SJ, RN, FO'C, JQ, MS, RT, MD, JL, AM, SY, MB, and BZ received honoraria and support for the travels from Novartis. VC received personal fees for consulting, lecture fees, and travel from Actelion, Bayer, Biogen Idec, Boehringer Ingelheim, Gilead, GSK, MSD, Novartis, Pfizer, Roche, Sanofi; grants from Actelion, Boehringer Ingelheim, GSK, Pfizer, Roche; personal fees for developing educational material from Boehringer Ingelheim and Roche. PV has been on the study steering group of the EXIST-1, 2, and 3 studies sponsored by Novartis, and co-PI on two investigator-initiated studies part-funded by Novartis. RN received grant support, paid to her institution, from Eisai and lectures fees from Nutricia, Eisai, Advicenne, and GW Pharma. YT received personal fee from Novartis for lecture and for copyright of referential figures from the journals, and received grant from Japanese government for intractable epilepsy research. SJ was partly financed by the EC Seventh Framework Programme (FP7/2007-2013; EPISTOP, grant agreement no. 602391), the Polish Ministerial funds for science (years 2013-2018) for the implementation of international cofinanced project and the grant EPIMARKER of the Polish National Center for Research and Development No STRATEGMED3/306306/4/2016. JK, PC, CH, JL, and JQ received research grant from Novartis. RM, LD'A, and SS are employees of Novartis. This study was funded by Novartis Pharma AG. The remaining authors declare that the research was conducted in the absence of any commercial or financial relationships that could be construed as a potential conflict of interest.
