# Supplementary material for: Burden of Illness and Quality of Life in Tuberous Sclerosis Complex: Findings From the TOSCA Study
Source: Front Neurol. 2020 Aug 28;11:904. doi: 10.3389/fneur.2020.00904 (PMC7485558; doi:10.3389/fneur.2020.00904)
Supplement: Supplementary file 1 [file Data_Sheet_1.PDF]

Supplementary Material

A. QUESTIONNAIRE FOR CHILDREN AND ADOLESCENT

**Section 1: Patient Assistance/Rights**		
**Q1. What is your relationship to the Tuberous Sclerosis Complex (TSC) patient?**		
Father	☐	
Mother	☐	
Other family relative	☐	
Other	☐	
**Q2. Does your child have any of the following when at home?**		
	**Yes**	**No**
Nurse assistance	☐	☐
Daily assistance by a professional carer (paid for)	☐	☐
Caregiver assistance from friend/family/relative (not paid for)	☐	☐
**Q3. Do you feel you receive enough assistance and support at home?**		
Yes	☐	
No	☐	
**Q4. Do you receive any of the following?**		
	**Yes**	**No**
Disability Allowance	☐	☐
Caregiver Allowance	☐	☐
Psychological Counselling	☐	☐
Social Worker Assistance	☐	☐
Social Services Support	☐	☐
Support in Completing Paperwork Regarding Benefits Available to you	☐	☐
**Q5a. Do you have:**		
Private Insurance	☐	(Go to Q5b)
Public Insurance	☐	(Go to Q6)
No Insurance	☐	(Go to Q6)
Not Applicable	☐	(Go to Q6)
**Q5b. If private insurance, do you feel you had to pay an additional premium due to your child's TSC condition?**		
Yes	☐	
No	☐	
**Q6. Have you been denied health or any kind of insurance due to your child's TSC?**		
Yes	☐	
No	☐	
**Q7a. Genetic Testing: Please tick the statement that applies to you:**		
We have had genetic testing for TSC	☐	(Go to Q7b)
We have been offered genetic testing but have not had this test conducted	☐	(Go to Q8)
We have not been offered genetic testing for TSC	☐	(Go to Q8)
**Q7b. If applicable, approximately how long did it take to receive the results following genetic testing?**		
	___________**Weeks**	
**Q8. Genetic Counselling: Please tick the statement that applies to you:**		
I have had genetic counselling	☐	
I have been offered genetic counselling but decided not to have this	☐	
I have not been offered genetic counselling for TSC	☐	
**Q9. If applicable, approximately how long was it between the following activities;**		
Diagnosis to genetic testing	__________**Months**	
Diagnosis to genetic counselling	__________**Months**	

Section 2: You and Your Treatment for Tuberous Sclerosis Complex (TSC)

Q1. How many different doctors manage your child's TSC?

1 doctor	☐	
2 doctors	☐	
3 doctors	☐	
More than 3 doctors	☐	

Q2a. Is your child's TSC managed by a: (tick all that apply)

General Practitioner/Family Doctor	☐	(Go to Q3a)
TSC Specialist (a doctor who is a medical expert in TSC)	☐	(Go to Q2b)
Other Speciality (e.g. Neurologist, Nephrologist, Neuro-pead, etc)	☐	(Go to Q3a)

Q2b. If managed by a TSC Specialist: Did you search for the specialist yourself?

Yes	☐	(If Yes, Go to Q2c)
No	☐	(Go to Q3a)

Q2c. If yes: Was it difficult to find a qualified TSC specialist?

Yes	☐	
No	☐	

Q3a. Do you have access to a TSC clinic when required? (i.e. a centre specialised for most problems of TSC)

Yes	☐	(If Yes, Go to Q3b)
No	☐	(Go to Q3a)

Q3b. If YES: How far away is the TSC clinic from your home:

Less than 50km from home	☐	
More than 50km from home	☐	

Q4. How satisfied are you with the following aspects of your child's treatment…

	Very Dissatisfied 1	Quite Dissatisfied 2	Quite Satisfied 3	Very Satisfied 4	Don't Know 5
Your level of involvement in treatment decisions	☐	☐	☐	☐	☐
How hospital visits regarding the various aspects of your child's TSC condition are co- ordinated	☐	☐	☐	☐	☐
How well different specialists managing your child's TSC condition communicate with each other	☐	☐	☐	☐	☐
That all aspects of your child's TSC condition are being managed	☐	☐	☐	☐	☐
That your child is being treated in accordance with national TSC guidelines	☐	☐	☐	☐	☐
How treatment options offered to your child are explained	☐	☐	☐	☐	☐
Getting drugs prescribed that you feel your child needs	☐	☐	☐	☐	☐
How your child's TSC condition is explained to you	☐	☐	☐	☐	☐
Your child's medical care for TSC	☐	☐	☐	☐	☐

Section 3: Social Support Services

Q1a. Which of the following sources of information have you used to learn more about your rights and benefits? Please tick all that apply.

	**Source used**
Social Worker	☐
Local Government	☐
Nurse	☐
Patient Group	☐
Doctor	☐
Internet / Websites	☐

Q1b. Please identify from the sources of information you use, which you find most useful ? Please tick one only

	**Source most useful**
Social Worker	☐
Local Government	☐
Nurse	☐
Patient Group	☐
Doctor	☐
Internet / Websites	☐

Q2. Which of the following best represents how you feel about the rights and benefits you have?

I feel well informed about my rights and benefits	☐
I do not feel very well informed about my rights and benefits	☐

Q3. Do you feel Social Services give you adequate support and information, concerning rights and benefits in your area?

Yes	☐
No	☐
Not Applicable	☐

Q4. TSC Association:

Are you in contact with the national TSC Association?

Yes	☐	(Go to Q5)
No	☐	(Go to next section)

Q5. Who put you in contact initially with the national TSC Association?

Doctor	☐
Nurse	☐
Other Healthcare Person	☐
Other Patient	☐
Family Member	☐
Internet	☐
Other	☐
Not Applicable	☐

Section 4: Clinical Trial Experience

Q1. Are you involved in any clinical trails concerning TSC? (By clinical trial we mean a scientific study of how a new medicine or treatment works in people)

Yes	☐	Continue to Q2
No	☐	Go to next section

Q2. Thinking about your experience with that clinical trial:

How often are you required to visit the trial centre for tests etc?

Less than once a month	☐
Approximately once a month	☐
Approximately twice a month	☐
Approximately three times a month	☐
Approximately four times a month	☐
More than four times a month	☐

Q3. Do you:

	**Yes**	**No**
a) Require an overnight stay when visiting the trial centre due to distance from your home	☐	☐
b) Receive time off work to attend the trial	☐	☐
c) Feel adequately involved in decisions	☐	☐
d) Feel your mobility needs have been taken into account (e.g. wheelchair, access, travel, etc.)	☐	☐

Section 5: Education

Q1. Is your child in mainstream education?

Yes	☐	Continue
No	☐	Go to Q5

Q2a. If your child is in mainstream education, does your child receive additional support in class?

Yes	☐	Go to Q2b
No	☐	Go to Q3

Q2b. If yes, does this cause your child any additional problems? (e.g. excluded from certain activities if support not available)

Yes	☐
No	☐

Q3. Are education programmes aimed at your child's difficulties available (e.g. Programmes designed for intellectual impairment and/or autistic spectrum disorder)?

Yes	☐
No	☐
Don't know	☐

Q4. Please indicate how strongly you agree or disagree with the following statements:

	Very Dissatisfied 1	Quite Dissatisfied 2	Quite Satisfied 3	Very Satisfied 4	Don't Know 5
My child is made to feel different because of their TSC	☐	☐	☐	☐	☐
I have sufficient dialogue with the school system	☐	☐	☐	☐	☐
There is adequate communication with all parties involved (e.g. parents, teachers, doctors)	☐	☐	☐	☐	☐
My child's education needs are adequately met	☐	☐	☐	☐	☐
My child's requirements have been taken into consideration	☐	☐	☐	☐	☐

Q5. Is your child schooled at home?

Yes	☐	Continue
No	☐	Go to Q7

Q6. Please indicate how strongly you agree or disagree with the following statements:

	Strongly Disagree 1	Slightly Disagree 2	Neither Agree or Disagree 3	Slightly Agree 4	Strongly Agree 5
I have sufficient dialogue with the home system	☐	☐	☐	☐	☐
There is adequate communication with all parties involved (e.g. parents, teachers, doctors)	☐	☐	☐	☐	☐
My child's education needs are adequately met	☐	☐	☐	☐	☐
My child's requirements have been taken into consideration	☐	☐	☐	☐	☐

Q7. If your child is less than 4 years old, Do you receive support in looking after your child?

Yes	☐
No	☐

Q8. All to Complete

Which of the following, if any, are available in your area? (Tick all that apply)

		If yes, are these services free to you?	
	**Yes**	**Yes**	**No**
Respite centres	☐ →	☐	☐
Independent living programmes	☐ →	☐	☐
Special holidays	☐ →	☐	☐
None of the above available	☐		

Section 6: Impact of TSC on Self and Family

Q1. What is your current employment status?

Employed (either full or part-time)	☐
Unable to work due to condition	☐
Unable to work but not due to child's condition	☐
Student	☐
Homemaker	☐

Q2a. Do you feel your career/education has been impacted by your child's TSC condition?

Yes	☐
No	☐

Q2b. If yes, in which of the following ways (please tick all that apply):

Career progression/promotions	☐
Choice of career	☐
Loss of employment	☐
Part-time work rather than full time	☐
Education level attained	☐

Q3. If currently working:

Please estimate the time off work in the last year due to your child's:

a) Ill-health

b) Hospital visits

Number of days per year	**Ill Health (Tick One)**	**Hospital Visits (Tick One)**
1-10 days	☐	☐
11-20 days	☐	☐
21-30 days	☐	☐
31-60 days	☐	☐
61-90 days	☐	☐
More than 90 days	☐	☐

Q4a. Do you feel your relationships have been impacted by your child's TSC condition?

Yes	☐	(Go to Q4b)
No	☐	(Go to Q5a)

Q4b. If yes, in which of the following relationships (please tick all that apply):

Family relationships	☐
Social relationships	☐
Work colleague relationships	☐

Q5a. What is the total annual income of your household?

☐ No income	☐ €30,000 - €39,999
☐ Under €10,000	☐ €40,000 - €50,000
☐ €10,000 - €19,999	☐ Over €50,000
☐ €20,000 - €29,999	☐ I prefer not to answer

Q5b Please estimate how much you spend in an average year on the following: (If you do not spend any money on these, please write in "0")

Medication (both prescription and bought directly from the pharmacy)	€____________
Travel to and from TSC Centre	€____________
Tests/Disease Assessments	€____________

B. QUESTIONNAIRE FOR ADULTS

**Section 1: Patient Assistance/Rights**		
**Q1. Home circumstances:**		
Lives alone	☐	
Lives with friends	☐	
Lives with spouse/partner	☐	
Lives with other family	☐	
**Q2. Do you need help with your daily activities, such as washing, dressing, eating etc?**		
Yes	☐	
No	☐	
**Q3. Do you have any of the following when at home?**		
	**Yes**	**No**
Nurse assistance	☐	☐
Daily assistance by a professional carer (paid for)	☐	☐
Caregiver assistance from friend/family/relative (not paid for)	☐	☐
**Q4. Do you feel you receive enough assistance and support at home?**		
Yes	☐	
No	☐	
**Q5. Do you receive any of the following?**		
	**Yes**	**No**
Disability Allowance	☐	☐
Caregiver Allowance	☐	☐
Psychological Counselling	☐	☐
Social Worker Assistance	☐	☐
Social Services Support	☐	☐
Support In Completing Paperwork Regarding Benefits Available to you	☐	☐
**Q6a. Do you have:**		
Private Insurance	☐	(Go to Q6b)
Public Insurance	☐	(Go to Q7)
No Insurance	☐	(Go to Q7)
Not Applicable	☐	(Go to Q7)
**Q6b. If private insurance, do you feel you had to pay an additional premium due to your Tuberous Sclerosis Complex (TSC) condition?**		
Yes	☐	
No	☐	
**Q7. Have you been denied health or any kind of insurance due to being or having a family member affected with TSC?**		
Yes	☐	
No	☐	
**Q8a. Genetic Testing: Please tick the statement that applies to you:**		
I have had genetic testing for TSC	☐	(Go to Q8b)
I have been offered genetic testing but have not had this test conducted	☐	(Go to Q9)
I have not been offered genetic testing for TSC	☐	(Go to Q9)
**Q8b. If applicable, approximately how long did it take to receive the results following genetic testing?**		
	___________**Weeks**	
**Q9. Genetic Counselling: Please tick the statement that applies to you:**		
I have had genetic counselling	☐	
I have been offered genetic counselling but have it	☐	
I have not been offered genetic counselling for TSC	☐	
**Q10. If applicable, approximately how long was it between the following activities**		
Diagnosis to genetic testing	___________**Months**	
Diagnosis to genetic counselling	___________**Months**	

Section 2: You and Your Treatment for Tuberous Sclerosis Complex (TSC)

Q1. How many different doctors manage your child's TSC?

1 doctor	☐	
2 doctors	☐	
3 doctors	☐	
More than 3 doctors	☐	

Q2a. Is your child's TSC managed by a: (tick all that apply)

General Practitioner/Family Doctor	☐	(Go to Q3a)
TSC Specialist (a doctor who is a medical expert in TSC)	☐	(Go to Q2b)
Other Speciality (e.g. Neurologist, Nephrologist, Neuro-pead, etc)	☐	(Go to Q3a)

Q2b. If managed by a TSC Specialist: Did you search for the specialist yourself?

Yes	☐	(If Yes, Go to Q2c)
No	☐	(Go to Q3a)

Q2c. If yes: Was it difficult to find a qualified TSC specialist?

Yes	☐	
No	☐	

Q3a. Do you have access to a TSC clinic when required? (i.e. a centre specialised for most problems of TSC)

Yes	☐	(If Yes, Go to Q3b)
No	☐	(Go to Q3a)

Q3b. If YES: How far away is the TSC clinic from your home:

Less than 50km from home	☐	
More than 50km from home	☐	

Q4. How satisfied are you with the following aspects of your child's treatment…

	Very Dissatisfied 1	Quite Dissatisfied 2	Quite Satisfied 3	Very Satisfied 4	Don't Know 5
Your level of involvement in treatment decisions	☐	☐	☐	☐	☐
How hospital visits regarding the various aspects of your TSC condition are co- ordinated	☐	☐	☐	☐	☐
How well different specialists managing your TSC condition communicate with each other	☐	☐	☐	☐	☐
That all aspects of your TSC condition are being managed	☐	☐	☐	☐	☐
That you are being treated in accordance with national TSC guidelines	☐	☐	☐	☐	☐
How treatment options offered to you are explained	☐	☐	☐	☐	☐
Getting drugs prescribed that you feel you feel you need	☐	☐	☐	☐	☐
How your TSC condition is explained to you	☐	☐	☐	☐	☐
Your medical care for TSC	☐	☐	☐	☐	☐

Section 3: Social Support Services

Q1a. Which of the following sources of information have you used to learn more about your rights and benefits? Please tick all that apply.

	**Source used**
Social Worker	☐
Local Government	☐
Nurse	☐
Patient Group	☐
Doctor	☐
Internet / Websites	☐

Q1b. Please identify from the sources of information you use, which you find most useful ? Please tick one only

	**Source most useful**
Social Worker	☐
Local Government	☐
Nurse	☐
Patient Group	☐
Doctor	☐
Internet / Websites	☐

Q2. Which of the following best represents how you feel about the rights and benefits you have?

I feel well informed about my rights and benefits	☐
I do not feel very well informed about my rights and benefits	☐

Q3. Do you feel Social Services give you adequate support and information, concerning rights and benefits in your area?

Yes	☐
No	☐
Not Applicable	☐

Q4. TSC Association:

Are you in contact with the national TSC Association?

Yes	☐	(Go to Q5)
No	☐	(Go to next section)

Q5. Who put you in contact initially with the national TSC Association?

Doctor	☐
Nurse	☐
Other Healthcare Person	☐
Other Patient	☐
Family Member	☐
Internet	☐
Other	☐
Not Applicable	☐

Section 4: Clinical Trial Experience

Q1. Are you involved in any clinical trails concerning TSC? (By clinical trial we mean a scientific study of how a new medicine or treatment works in people)

Yes	☐	Continue to Q2
No	☐	Go to next section

Q2. Thinking about your experience with that clinical trial:

How often are you required to visit the trial centre for tests etc?

Less than once a month	☐
Approximately once a month	☐
Approximately twice a month	☐
Approximately three times a month	☐
Approximately four times a month	☐
More than four times a month	☐

Q3. Do you:

	**Yes**	**No**
a) Require an overnight stay when visiting the trial centre due to distance from your home	☐	☐
b) Receive time off work to attend the trial	☐	☐
c) Feel adequately involved in decisions	☐	☐
d) Feel your mobility needs have been taken into account (e.g. wheelchair, access, travel, etc.)	☐	☐

Section 5: Impact of TSC on Self and Family

Q1. What is your current employment status?

Employed (either full or part-time)	☐	Continue
Unable to work due to condition	☐	Go to Q5
Unable to work but not due to child's condition	☐	
Student	☐	
Homemaker	☐	

Q2a. Do you feel your career/education has been impacted by your child's TSC condition?

Yes	☐
No	☐

Q2b. If yes, in which of the following ways (please tick all that apply):

Career progression/promotions	☐
Choice of career	☐
Loss of employment	☐
Part-time work rather than full time	☐
Education level attained	☐

Q3. If currently working:
Please estimate the time off work in the last year due to your child's:
a) Ill-health
b) Hospital visits

Number of days per year	**Ill Health (Tick One)**	**Hospital Visits (Tick One)**
1-10 days	☐	☐
11-20 days	☐	☐
21-30 days	☐	☐
31-60 days	☐	☐
61-90 days	☐	☐
More than 90 days	☐	☐

Q4a. Do you feel the career/education of other members of your family have been impacted by your TSC condition?

Yes	☐
No	☐

Q4b. If yes, in which of the following ways (please tick all that apply):

Career progression/promotions	☐
Choice of career	☐
Loss of employment	☐
Part-time work rather than full time	☐
Education level attained	☐

Q5a. Do you feel your relationships have been impacted by your child's TSC condition?

Yes	☐	(Go to Q5b)
No	☐	(Go to Q6a)

Q5b. If yes, in which of the following relationships (please tick all that apply):

Family relationships	☐
Social relationships	☐
Work colleague relationships	☐

Q6a. Is adult care for patients with TSC available in your area?

Yes	☐
No	☐

Q6b. If No, Are you under paediatric care?

Yes	☐
No	☐

Q7a. In your experience was there a smooth transition from paediatric care to adult care?

Yes	☐	
No	☐	
Don't Know / Not Applicable	☐	

Q7b. Did you have adequate support for the transition from paediatric care to adult care?

Yes	☐	(Go to Q7c)
No	☐	(Go to Q8a)

Q7c. If yes, Were they free?

Yes	☐	(Go to Q8a)
No	☐	(Go to Q7d)

Q7d. If no, were you entitled to reimbursements?

Yes	☐	
No	☐	
Not Applicable	☐	

Q8a. What is the total annual income of your household?

☐ No income	☐ €30,000 - €39,999
☐ Under €10,000	☐ €40,000 - €50,000
☐ €10,000 - €19,999	☐ Over €50,000
☐ €20,000 - €29,999	☐ I prefer not to answer

Q8b Please estimate how much you spend in an average year on the following: (If you do not spend any money on these, please write in "0")

Medication (both prescription and bought directly from the pharmacy)	€____________
Travel to and from TSC Centre	€____________
Tests/Disease Assessments	€____________

Q9a. Do you suffer from facial angiofibromas (a rash of reddish spots or bumps on the nose and cheek)?

Yes	☐	Go to Q9b
No	☐	Go to Q10

Q9b. Do your facial angiofibromas impact on your quality of life?

Yes	☐	Go to Q9c
No	☐	Go to Q10

Q9c. How much do your facial angiofibromas impact on your quality of life?

Low impact	☐
Medium impact	☐
High impact	☐

Q10. Do you have epilepsy?

Yes ☐ Please complete the following questionnaire

No ☐ Thank you for completing the questionnaire. You do not need to complete the next section
